# Molecular and immunological studies on *Theileria equi* and its vector in Egypt

**DOI:** 10.1007/s10493-024-00933-4

**Published:** 2024-07-05

**Authors:** Reem M. Ramadan, Noha Madbouly Taha, Hend M. Auda, Eslam M. Elsamman, Mohamed M. El-Bahy, Mai A. Salem

**Affiliations:** 1 Department of Parasitology, Faculty of Veterinary Medicine, Cairo University, Giza, 12211 Egypt; 2 Department of Parasitology, Faculty of Medicine, Cairo University, Giza, Egypt; 3 Department of Medicine and Infectious Diseases, Faculty of Veterinary Medicine, Cairo University, Giza, Egypt; 4 Faculty of Veterinary Medicine, Cairo University (Equine Veterinarian), Giza, 12211 Egypt

**Keywords:** *T. equi*, *Hyalomma*, *Rhipicephalus*, Phylogenic, Oxidative stress, Gene expression

## Abstract

Equine piroplasmosis is not fully understood regarding pathogenicity, prophylaxis, host immune response expression, and specific vectors. Accurately identifying the parasite vector is crucial for developing an effective control plan for a particular infection. This study focused on morphologically identifying two *Hyalomma* species (*H. anatolicum* and *H. marginatum*) and one *Rhipicephalus annulatus* (*R. annulatus*) at the species level. The identification process was followed by phylogenetic analysis using the neighbor-joining method based on the cytochrome oxidase subunit 1 (COXI) gene as a specific vector for *Theileria equi* (*T. equi*) in horses. *T. equi* was diagnosed morphologically and molecularly from infected blood samples and crushed tick species using conventional PCR. Subsequently, phylogenetic analysis based on the amplification of the 18 S rRNA gene was conducted. The obtained sequence data were evaluated and registered in GenBank under accession numbers OR064161, OR067911, OR187727, and OR068139, representing the three tick species and the isolated *T. equi*, respectively. The study demonstrated that *T. equi* infection leads to immune system suppression by significantly increasing the levels of oxidative stress markers (CAT, GPx, MDA, and SOD) (*P* ≤ 0.0001), with this elevation being directly proportional to parasitemia levels in infected blood cells. Furthermore, a correlation was observed between parasitemia levels and the expression of immune response infection genes (IFN-gamma, TGF-β1, and IL-1β cytokines) in infected horses compared to non-infected equine. Common macroscopic symptoms indicating *T. equi* infection in horses include intermittent fever, enlarged lymph nodes (LN), and tick infestation.

## Introduction

Horses, mules, donkeys, and ponies are globally distributed animals whose value varies from country to country. They serve multiple purposes in Egypt, including public transport, riding, and racing. Additionally, they have a significant economic impact on their owners, particularly in rural areas, as they are used to transport agricultural products and utensils along narrow roads between cultivated fields.

Equines affected by several diseases often exhibit non-specific symptoms such as fever, anemia, and edema, which can be associated with conditions like anaplasmosis, dourine, lyme disease, leptospirosis, West Nile fever, trypanosomiosis (surra) and equine piroplasmosis (Sazmand et al. 2020). Hemoglobinuria is characteristic of piroplasmosis infection but may occur due to toxicity or metabolic disorders (Rothschild 2013). Jaundice can be a symptom of anaplasmosis, leptospirosis, surra, and equine piroplasmosis. Additionally, infections by hard ticks can be associated with piroplasmosis and Lyme disease and can cause hind-limb paralysis (Mahmoud et al. 2020). Under these circumstances, multiple symptoms such as fever, anemia, tick infection, and hemoglobinuria, particularly in older animals during summer, increase the likelihood of piroplasmosis infection (Almazán et al. 2022).

*Theileria equi* is a crucial intracellular hemoprotozoan causing piroplasmosis in equines and is transmitted by various hard ticks (Scoles and Ueti 2015). Previously considered a species of *Babesia*, detailed epidemiological investigations by Mehlhorn and Schein (1998) reclassified it as *Theileria*. There remains a lack of knowledge about the specific tick vector for *T. equi*, its adverse effects on the host immune system, and its accurate genotyping concerning the parasite and its tick vector. Several studies have conducted serological tests, but these often lack sensitivity in cases of chronic infection or following treatment (Mahmoud et al. 2016; El Akkad et al. 2022).

Accurate identification of the parasite and its vectors through morphological analysis alone may be insufficient due to the high morphological similarity between closely related species (Ashour et al. 2023a). In this context, molecular identification-based techniques offer a precise and accurate method for diagnosing several parasitic diseases, including babesiosis, particularly in the subclinical phase (Jaffer et al. 2010).

Parasitic infections cause varying degrees of stress, including oxidative stress, as hosts produce reactive oxygen species (ROS) to combat invasive pathogens. However, these chemicals cannot discriminate between infectious agents and host cells (Esmaeilnejad et al. 2014). Oxidative stress occurs when ROS overwhelm the host’s defenses (Rahal et al. 2014). Overproduction of ROS can induce oxidative changes in DNA, proteins, and lipids, which are essential cell components. ROS-induced lipid peroxidation of cell membranes leads to loss of selective permeability and increased cellular damage. Oxidative DNA damage can result in mutations, replication errors, genomic instability, and cell death, while oxidative protein damage can lead to the loss of metabolic activities (Klaunig et al. 2010).

The cell-mediated immune response is crucial for defending the host against infection by reducing pathogen multiplication, though it may cause pathology. T helper 1 (Th1) cytokine, IFN-gamma, plays an essential role against intracellular parasites during the early acute stages of disease (Robb & Hill 2012). IFN-gamma is vital for inducing host immunity, controlling the production of IgG2 from bovine B-cells, and stimulating monocytes for enhanced microbicidal activity. However, this shift likely results from an antagonistic immunological response to the Th1 response. Numerous studies have examined the impact of IFN-gamma, IL-1β, and TGF-β1 production during the acute stage of infection (Namangala et al. 2009).

*T. equi* develops in lymphoid cells and erythrocytes to complete its life cycle in the mammalian host. After entering the host’s mononuclear cells, *Theileria* sporozoites differentiate into the macro-schizont stage, triggering the host’s defensive immunological responses. The primary immune mechanism targets lymphocytes infected with schizonts and is cell-mediated. Mononuclear cells (lymphocytes and macrophages) release cytokines such as interferon (IFN), tumor necrosis factor (TNF), and interleukins (ILs) to inhibit parasite growth. Over time, numerous investigations have examined the role of cytokines in ruminant piroplasmosis; however, few studies have concerned the evaluation of inflammatory mediators in equine theileriosis (Mostafavi et al. 2020).

The current study was carried out for morpho-molecular identification of ticks infecting equines in Egypt. Detection of blood parasites infecting equines suffering from hard tick infestation and assessment of oxidative stress and immunological markers and their significance in infected horses.

## Materials and methods

### Area of the study and animals

A private equine farm in Nazlet El-Saman Giza (31.208853 longitude & 30.013056 latitudes), Egypt, specialized for the production of light-riding Arabian horse types. The farm is composed of two wings combined in a single 1000 m^2^ yard with trees and a number of large and normal individual stables. One wing of the farm contained 75 animals while the other for housing of 50 animals. Their ages vary from newly born foals up to 16 years old, contain ♂ & ♀ in an average of one ♂ to > 10 ♀. The females were inseminated artificially, the foal still with its mare and gradually separated from its mother during 1–2 weeks before its weaning age which occurs at 4–6 months age according to the purpose in which the new foal will be used and the general health condition of their mare. They feed them by balanced rations composed of hay (hay cubes or hay-based pellets) by a percentage of 1% of their body weight, with concentrates (barley, corn grains) by a percentage of 1.5-2% of its body weight, carrots, vegetable clover, dried whey and linseed, were used also. Moreover, commercial horse feed and mineral additives include Tribute High fat pelleted containing 13% fat, 20% fibers & low starch and sugar (15%) *(TRBUTE*^*R*^ Equine nutrition*)* and Biotinvet 10.000 dietary mineral feed for horses. (*EQUIPLANET*, Torreselle *di, Piombino Dese (PD) Italy)* with water ad-libitum. Foals were vaccinated against Equine influenza and Equine herpes at 3 old months. No special regular medical investigation was applied on the farm. Other than routine deworming and external parasite eradication using specific drugs every 6 months. The owners invited a veterinarian only when he recognized the presence of special animal problems in this farm previously diagnosed as chronically infected by external parasites which usually increased during the warm months of the year.

The examined animals have several specific and non-specific symptoms and some of them are infected by hard ticks. The farm was investigated parasitologically and is still under observation for 6 successive months. During this period monthly identified blood and tick samples were collected and transferred directly to the Parasitology laboratory in the Faculty of Veterinary Medicine, Cairo University following Ashour et al. (2023a).

### Collection and examination of blood samples

Whole blood samples were collected from examined animals having clinical signs (75 samples) using jugular vein puncturing in Vacutainer tubes®. Part of the samples were collected with an anticoagulant (EDTA tube) and after that samples were transported to the lab using an icebox. Thin blood films were prepared using the EDTA non-coagulated samples on clean glass slides and stained with Giemsa to be examined using microscopic at 1000× magnification using oil immersion (Khalifa et al. 2023; El-Bahy et al., 2023). A number of 20 fields were analyzed in each stained slide. The diagnosed blood parasites were identified according to Nadal et al. (2022). In addition, EDTA non-coagulated blood was used for hematological assessment and extracting DNA using FTA® Elute cards (Whatman Cat. No. WB120410).

### Collection & identification of hard ticks

The approximate number and distribution of ticks on the infected horse body were determined. In Giza, ticks from 75 horses were collected and identified, and they were carefully collected in suitably ventilated tubes and transferred alive directly to the laboratory. Part of each sample was fixed in 70% ethyl alcohol and 20% glycerol and used for classification and identified morphologically after this using a stereomicroscope and taxonomic keys (Okely et al. 2021). The other part was kept for genotyping of ticks and tick-borne parasites.

### Genotyping study of ticks and the diagnosis of blood parasite

#### DNA extraction from blood and ticks

Seventy-five blood samples from infected equines as well as 880 tick samples that were diagnosed morphologically were inspected genetically. Ticks were crushed separately in a mortar into tiny fragments with liquid nitrogen. In accordance with the manufacturer’s guidelines, 200 µL of blood and tick samples were used to isolate genomic DNA using a Thermo Scientific GeneJET Genomic DNA Purification Kit (Thermo Fisher, Darmstadt, Germany). Before being used, genomic DNA was kept at -20 °C.

#### PCR amplification of targated genes of *T. Equi* parasites and ticks

By amplifying and sequencing the cytochrome oxidase c subunit I (COXI) gene, ticks were identified at the species level (Abdullah et al. 2022). The 18 S rRNA gene (small subunit ribosomal RNA) was amplified using PCR in order to identify *T. equi* from blood samples and the crushed ticks following the method described by Abdullah et al., (2022). 12.5 µL of the Cosmo Taq DNA Polymerase Master Mix (Willowfort, UK) in a total volume of 25 µL, 3 µL of the extracted DNA as a template, and 1 µL of 10 pmol of each primer were used in the amplification reaction (Ramadan et al. 2020). Table (1) provides specific primer and PCR conditions. The identical procedures were applied to both the negative and positive controls, which contained nuclease-free water and the genomic DNA of well-known blood parasites (Salem et al. 2023). The primers and experimental conditions are summarized in Table (1).

**Table 1:** Oligonucleotide primer pairs used in PCR amplifications for the detection of *T. equi* and different tick species of horses

Parasite	Target Gene (Amplicon size)	Primer sequence	Amplification conditions	Reference
*T. equi*	18 S rRNA (360)	F: 5’ CTTCAGCACCTTGAGAGAAATC 3’ R: 5’ TGCCTTAAACTTCCTTGCGAT 3’	94 °C for 7 min; 35 cycles (94 °C, 1 min; 54 °C, 1 min; 72 °C, 1 min.), 72 °C, 7 min	(Elsawy et al. 2021)
*R. annulatus*	COXI (1100)	F: 5’ CTGATATAGCTTTTCCACG 3’ R: 5’ CCTAAAATTGATGATGC 3’	95 °C for 2 min; 30 cycles (94 °C, 1 min; 65 °C, 1 min; 72 °C, 2 min.), 72 °C, 5 min	(Goolsby et al. 2016)
*H. anatolicum*	COXI (820)	F: 5’GGAACAATATATTTAATTTTTGG3’ R:5’ATCTATCCCTACTGTAAATATATG3’	94 °C, 5 min; 30 cycles (94 °C 1 min; 45 °C, 1 min; 72 °C, 1 min), 72 °C, 10 min	(Ashour et al. 2023b)
*H. marginatum*	COXI (860)	F: 5’ AATTTACAGTTTATCGCCT 3’ R: 5’ CATACAAT AAAGCCTAATA 3’	95 °C, 5 min; 32 cycles (92 °C 1 min; 46 °C, 35 s; 72 °C, 1.5 min), 72 °C, 7 min	(Vial et al. 2016)

F: forward, R: reverse, bp: base pair, min: minutes, sec: second

#### Sequencing & phylogenetic study

The QIAquick purification extraction kit (Qiagen, Hombrechtikon, Switzerland) was utilized to purify the PCR products, and the BigDye Terminator V3.1 sequencing kit (Applied Biosystems, Waltham, MA, USA) was utilized for sequencing (Ramadan et al. 2021). Using the ChromasPro program (ChromasPro 1.7, Technelysium Pty Ltd., Tewantin, Australia), the sequences were put together into contigs. The sequences were analyzed using BLAST and matched with reference sequences found in the GenBank (Taha et al. 2024a). Using 1000 bootstrap repeats, the Neighbor-joining method in MEGA 11 software (Maximum composite likelihood model) was used to create the phylogenetic trees.

### Measurements of oxidative stress markers

After examination of horses having clinical signs, they are divided into three groups of 25 animals according to the level infestation of ticks [tick density was calculated as numbers of ticks per 10^2^ inch (Tagliapietra et al. 2011)] and clinical signs; Low infection [1–2 ticks/10^2^ inch] (Horses with a mild tick infestation and no clinical signs), Moderate infection [3–5 ticks/10^2^ inch] (Horses with moderate tick infestation and mild clinical signs, High infection [more than 5 ticks/10^2^ inch] (Horses with severe tick infestation and severe clinical signs) and control negative healthy horses (*n* = 15) with no clinical signs of disease, no tick infestation and parasite-free blood and fecal samples.

Measurements of oxidative stress markers and cytokines expression quantification are performed on the various groups. The serum samples were evaluated for several indicators of oxidative stress. Malondialdehyde (MDA), Superoxide dismutases (SOD), catalase (CAT) and glutathione peroxidase (GPX) levels were measured in both positive and negative sera of examined horses using specialized kits (Salem et al. 2016).

### Cytokines expression quantification

#### Extraction of RNA

Using 8.5 µl of sterile TRIzol Reagent (Invitrogen Life Technologies, Carlsbad) and 10 pmol of Metabion, International AG, total RNA was extracted from the buffy coats of 75 horses. Two microliters of template RNA were utilized, in accordance with the manufacturer’s recommendations. To determine the quantity and integrity of RNA, an aliquot of total RNA diluted in RNase-free water was put away, and the remaining sample was maintained at -80 °C until gene expression analysis. Using a Nano-Drop (ND-1000 Spectro-photometer, Nano-Drop Technologies Inc, Delaware, USA), the concentration and purity of the PCR were ascertained (Salem et al. 2024).

#### Real-time PCR (RT-PCR)

Relative RT-PCR was carried out to measure the cytokine genes, IFN-gamma, IL-1β and TGF- β_1_. RT-PCR was done by the Cepheid SmartCycler® II system (Sunnyvale, CA, USA) using primers mentioned in Table (2). The reaction final volume was set up to 25 µl containing 0.5 µl of each primer (10 pmol), 12.5 µl SYBR Green PCR master mix (Applied Biosystems, USA), 1 µl cDNA (400ng) and 10.5 µl RNase free water. There were both positive and negative controls for every gene of interest. Following an initial incubation at 95 °C for 5 min, forty cycles of amplification were carried out for the glyceraldehyde-3-phosphate dehydrogenase (GADPH), IFN-gamma, TGF-β1 and IL-1β genes, with denaturation at 95 °C for 30 s, annealing at 60 °C for 30 s, and extension at 60 °C for 30 s.

**Table 2:** Oligonucleotide primer for RT-PCR

Gene	Primer sequence	Reference
IFN-gamma	F: 5-TCTTTAACAGCAGCACCAGCAA-3 R: 5-GCGCTGGACCTTCAGATCAT-3	Ainsworth et al. (2003)
TGF-β1	F: 5-TGACAGCAAAGATAACACACTCC-3 R:5-TCAATGGTGGCCAGATCA-3	Padoan et al. (2013)
IL-1β	F: 5-AGTCTTCAGTGCTCAGGTTTCTGA-3 R: 5-TGCCGCTGCAGTAAGTCATC − 3	Ross et al. (2012)
GADPH	F: 5-CCTGGAGAAACCTGCCAAGT-3 R:5-GCCAAATTCATTGTCGTACCA-3	Ross et al. (2012)

### Statistical analysis

The study data were tabulated in an Excel sheet. Numerical data were shown as mean ± standard deviation (Taha et al. 2024b). ANOVA followed by a post-hoc test was performed using SPSS (SPSS Inc., Chicago, IL, USA). *P*-value (≤ 0.0001) was considered statistically significant (Taha et al. 2022; Ramadan et al. 2023). The 2(-Delta Delta C(T) method was used to analyze the relative changes in gene expression from real-time quantitative PCR (Livak and Schmittgen 2001).

## Results

### Clinical signs in inspected equines

**Fig. 1:**
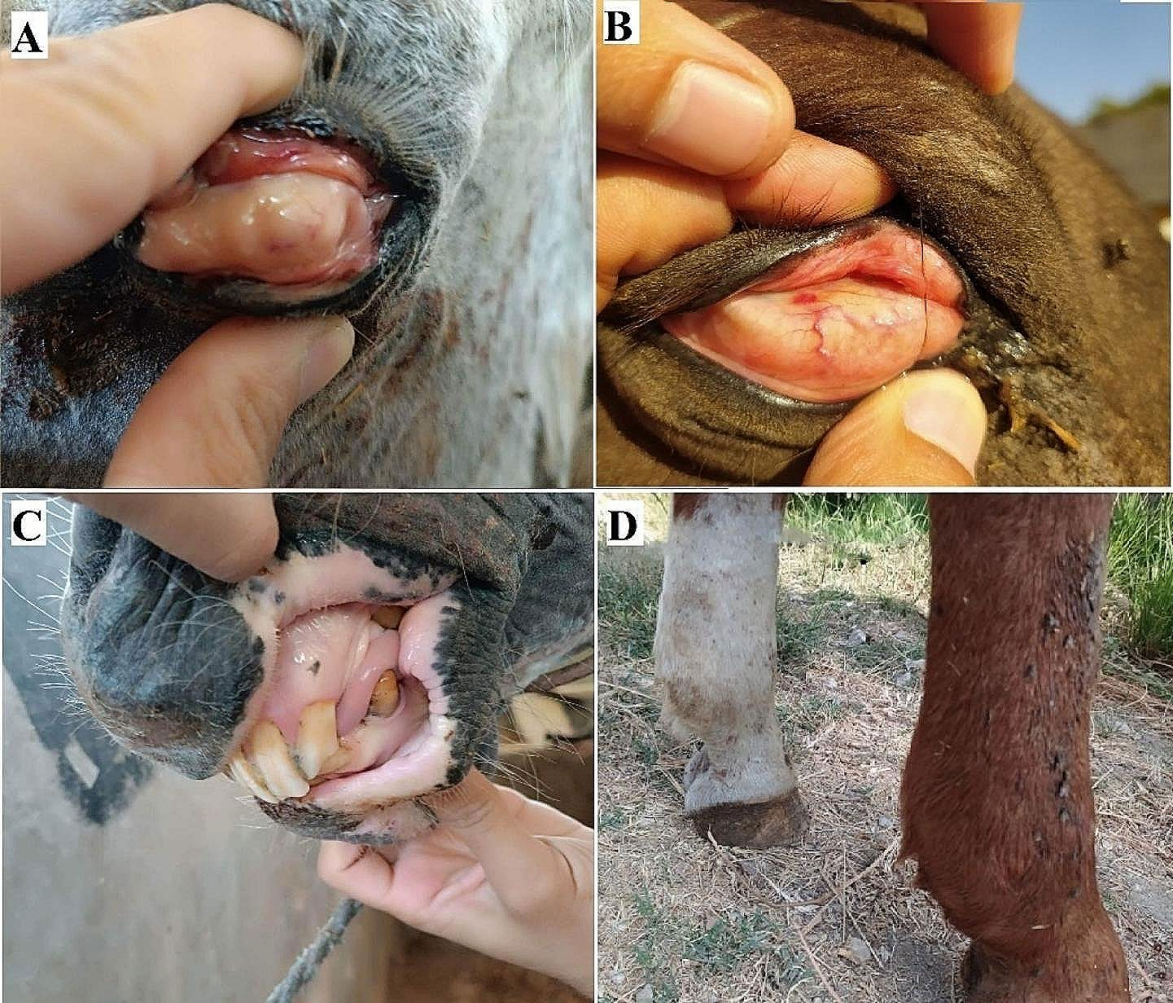
**A&B**) Peticial hemorrhage and icteric 3rd eyelid and conjunctiva. **C**) Pale (icteric) mucous membrane of the Gum. **D**) Edema of the distal limbs (Fetlock joint)

**Fig. 2:**
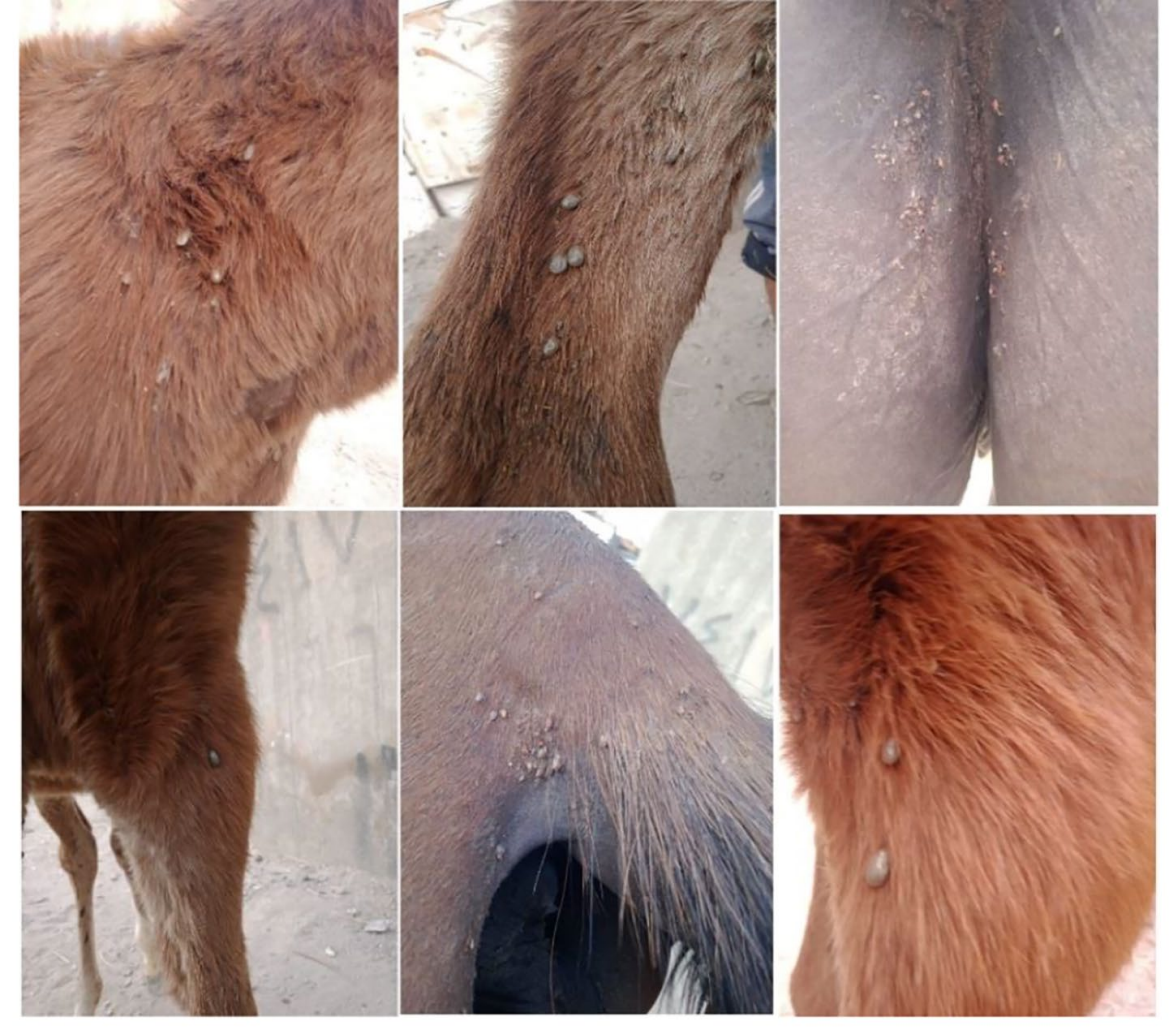
The spread of tick infestation across the entire body of horses on the outer & inner sides of the limbs, between the thigh and under the tail

Apparently, an inspection of the investigated equines revealed the presence of general symptoms of disease affection by different percentages as described in Table (3), The more prominent one is symptoms of anemia as pale mucus membrane was recorded in 25.33% followed by ecchymoses on the conjunctiva and congested mucous membranes with some petechial hemorrhages (17.33%) (Fig. 1). Hard tick infestation was detected in 21 animals (28%); 13 of them had enlargement in the pre-scapular lymph node, and 8 of them had edoema in the Fetlock joint and hemoglobinuria (Fig. 2). Other symptoms such as transitory fever, anorexia, colic, dyspnea, elevated respiratory, icterus and weight loss were recorded (Table 3).

**Table 3:** Clinical signs diagnosed in inspected equines concerning piroplasmosis infection in blood film

Diagnostic symptoms in 75 inspected horses	Incidence of symptom		Piroplasm in blood film	
	No.	%	No.	%
Transitory fever	9	12	9/9	100
Anorexia	11	14.66	5/11	45.45
Elevated respiratory	9	12	5/9	55.55
Colic	10	13.33	0/10	-
Weight loss	8	10.66	2/8	25.0
Pale mucous	19	25.33	15/19	78.95
Conjunctivitis	13	17.33	8/13	61.54
Icterus	9	12	4/9	44.44
hemoglobinuria	8	10.66	2/8	25.0
Enlarged Pre-Scapular L.N.	13	17.33	13/13	100
Edema in the Fetlock joint	8	10.66	8/8	100
Infestation by hard ticks	21	28	20/21	95.24

### Microscopic examination of blood smears

**Fig. 3:**
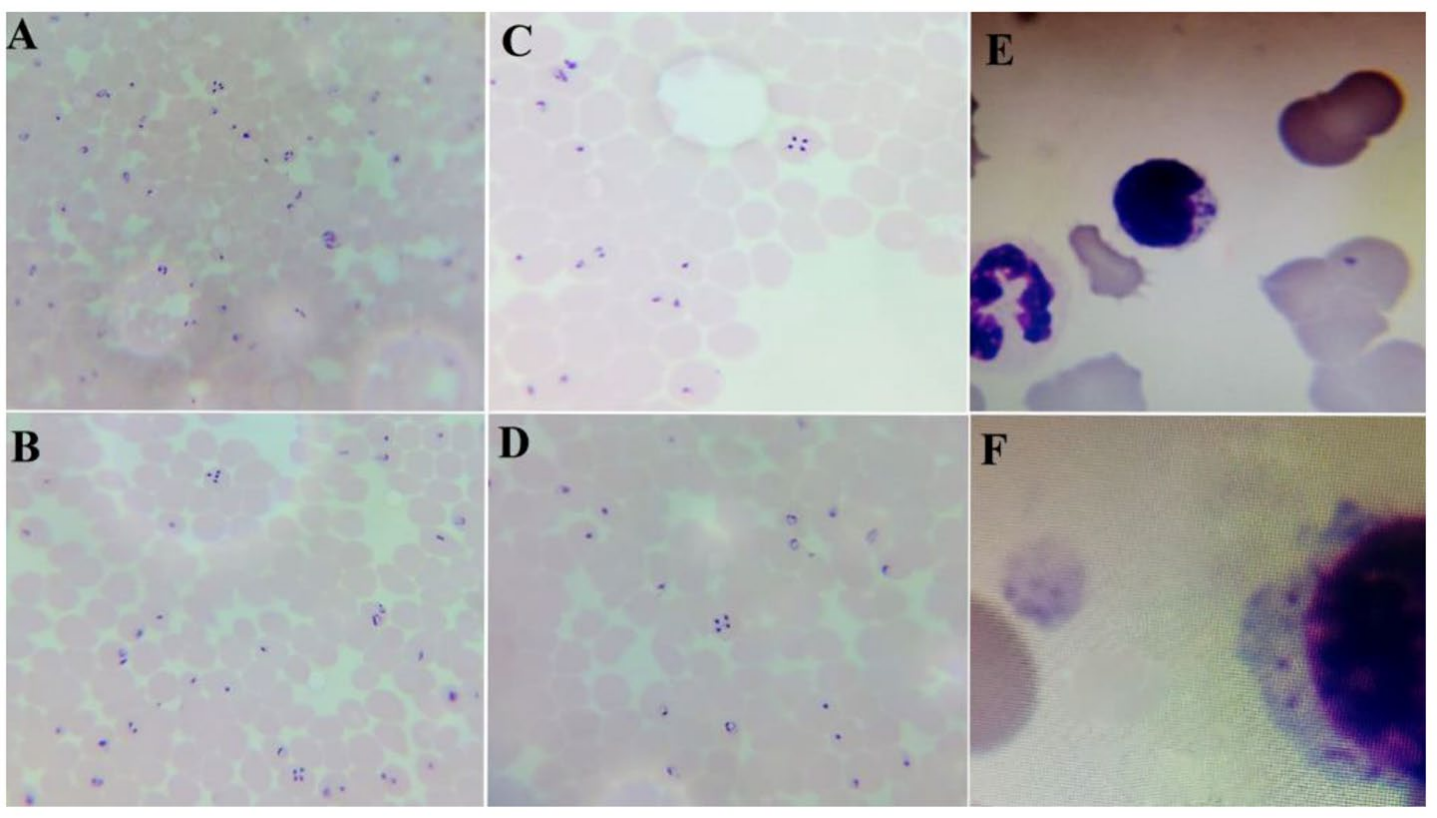
Different stages of *T. equi* in blood smears showing its characteristic Maltese cross-forms in RBCs, (**A, B, C, D)** and its Schizont in lymphocyte (**E, F)**, (Fixed blood film stained with Giemsa-under Oil immersion X100 magnification)

As the original symptoms suppose infection by blood parasites, microscopic examination of Giemsa-stained thin blood film from the infected animals revealed infection by the pear shape merozoites of *T. equi* (formerly *Babesia equi*) in a single form or in its characteristic tiny 4 pear-shaped structures that had been seen in Maltese cross-forms inside the RBCs as well as its characteristics schizont in lymphocytes, that appears as spherical bodies with chromatin granules and blue cytoplasm (Fig. 3).

### Relation between *T. Equi* in blood and the previously diagnosed symptoms

Linking the relation between all of the previous macroscopic symptoms and microscopic detection of *T. equi* in each blood film, the data in Table (3) revealed that there is a strong relation reaching 100% with symptoms such as Fever (exceeds 40 ^o^C), edema in the fetlock joint & hard tick infestation. A little lower association with other symptoms such as pale mucous, hemoglobinuria, conjunctivitis and elevated respiratory and transitory fever. The presence of the infection has a low association with other symptoms recorded in these animals such as colic, weight loss and icterus (Table 3).

### Ticks species isolated from equines

Morphological identification of collected samples from different animals during the study period revealed infestation by 2 species of *Hyalomma* (*H. anatolicum & H. marginatum*(Koch, 1844) as well as one species of *Rhipicephalus* (*R. annulatus*(Say, 1821).

#### *Hyalomma anatolicum*

The adult *H. anatolicum* is characterized by its pale/whitish legs, the mouth portion is long and front, and the female scutum is dark brown in color with smoothness at the rear margin. Female spiracles have a small dorsal prolongation compared to male spiracles, and the genital aperture is enlarged and may have a triangular or circular shape. The anterior genital opening is present. Palps and festoons are also present. The male genital aperture outline is rhomboidal in shape, distinctly longer than wide, straight to slightly arched anteriorly, with two posterior ridges, a banana-shaped spiracle, and three pairs of anal shields: adanal plates with squared ends, lateral grooves short, with a rough surface with paracentral festoons joined anteriorly forming arch, and the cervical grooves, which are evident and deep and half the length of the conscutum.

#### *Hyalomma marginatum*

The other species is *H. marginatum* (Koch, 1844) as it is morphologically identified according to the keys of Ros-García et al. (2013). The scutum was dark in colour, smooth, and had a clearly sinuous posterior border dorsally. The scutum’s posterior boundary was reached by the somewhat deep lateral and cervical grooves. The dorsal surface of the middle segments had vivid banding around the legs. The vestibular part of the vagina protruded somewhat from the large, shallow, and rounded (U-shaped) genital orifice on the ventral side. Long postero-lateral and postero-median spurs were present in Coxa I. A well-developed, roughly triangular postero-lateral spur was seen in Coxae II–IV. The large spiracular plates had scant circumspiracular setae and a perforated section of the dorsal prolongation. Their circumspiracular setae are scant, their lateral and cervical grooves are not punctated, and these characteristics set them apart from other *Hyalomma* species.

#### *Rhipicephalus annulatus*

Male *R. annulatus* (Say, 1821) characteristics include colour variations between yellow and brown, the absence of a caudal appendage, a circular spiracle without a dorsal prolongation, and two pairs of anal shields: elongate spindle-shaped adanal plates and supplementary adanal plates. The female had almost parallel sides and a rounded posterior edge tip. Her mouthparts were the same as those of males, and she had a dense covering of white setae. Her coxa 1 spurs were indistinct, her posterior margin was free of festoons, and her genital opening was semicircular. The infestation was distributed inside the ear, around the eyelids, on the inner sides of the legs, between the thigh and under the tail.

### Genotypic identification of the recorded tick vector

Based on sequence analysis of the positive PCR results of the COXI gene, tick species were categorized into two genera: *Hyalomma* and *Rhipicephalus*; this was confirmed using a sequence identity of 98 to 100% with tick species sequences in GenBank. *R. annulatus* was the most prevalent species of tick, accounting for 55.3%, followed by *H. marginatum* at 25.5%, and *H. anatolicum (H. anatolicum*) at 19.2% (Table 4). Table (5) lists the accession numbers of the COXI gene sequences of the detected ticks.

**Table 4:** Prevalence of the tick species

Tick Species	Number (%)
*R. annulatus*	487 (55.3)
*H. marginatum*	224 (25.5)
*H. anatolicum*	169 (19.2)
Total number collected	880

**Table 5:** The accession numbers of studied ticks & *T. equi*

Samples	Accession number	Source	% of identity	Target gene
*R. annulatus*	OR064161	Hard ticks	KX228542 (100%); AF132825 (98.68%)	COXI
*H. anatolicum*	OR067911		MH459377 (100%); MK462196 (99.55%)	
*H. marginatum*	OR187727		MZ687115 (100%); KU130611 (99.41%)	

### Genotypic identification of the diagnosed *T. equi* in equine blood samples and ticks

**Fig. 4:**
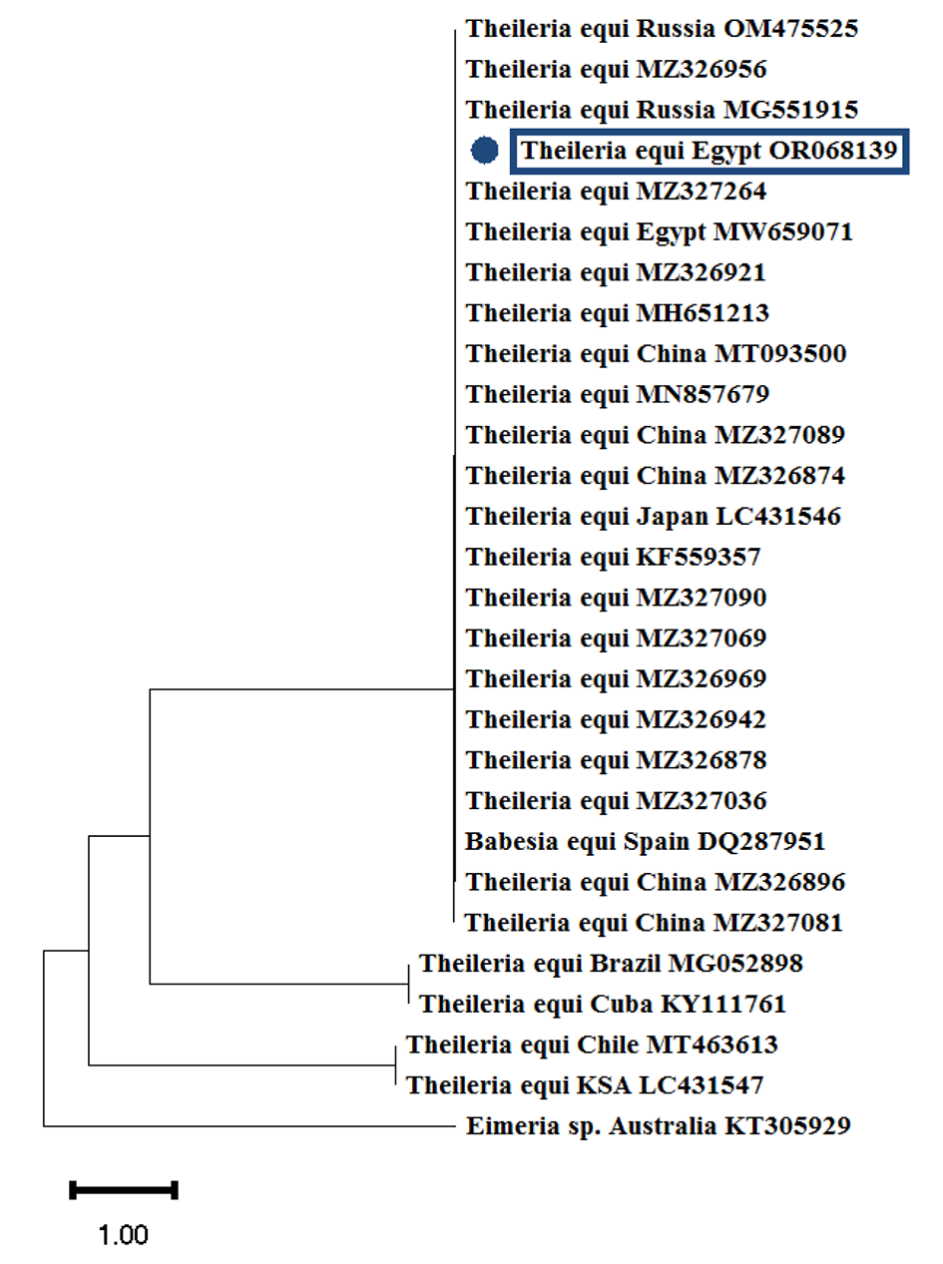
Phylogenetic analysis using the Neighbor-joining technique. The history of the evolution of *T. equi* isolates, based on the 18 S rRNA gene. This study’s accession numbers are those with blue dots. *Eimeria* sp. KT305929 gene was used as an outgroup. The number of nucleotide substitutions/sites is represented by a scale bar at the base of the tree

After molecular analysis of both blood and tick samples, the prevalence of *T. equi* in adult ticks was 43.9% (386/880), whereas it was 50.7% (38/75) in the horse blood specimens (Table 6). BLAST analysis of *Theileria* based on the 18 S rRNA gene was consistent with the sequence results (Fig. 4), identifying *T. equi* found in ticks and blood and deposited in GenBank under accession number (OR068139), showing high similarity with MZ326926 (100%) and ON429011 (99.54%).

**Table 6:** Prevalence of *T. equi* among the tick species

Tick Species	T. equi / Tick spp. (%)
*R. annulatus*	217 / 56.2%
*H. marginatum*	128 / 33.2%
*H. anatolicum*	41 / 24.3%
Total number collected	386 / 43.86%

### Oxidative stress of *T. Equi* on infected horses

**Fig. 5:**
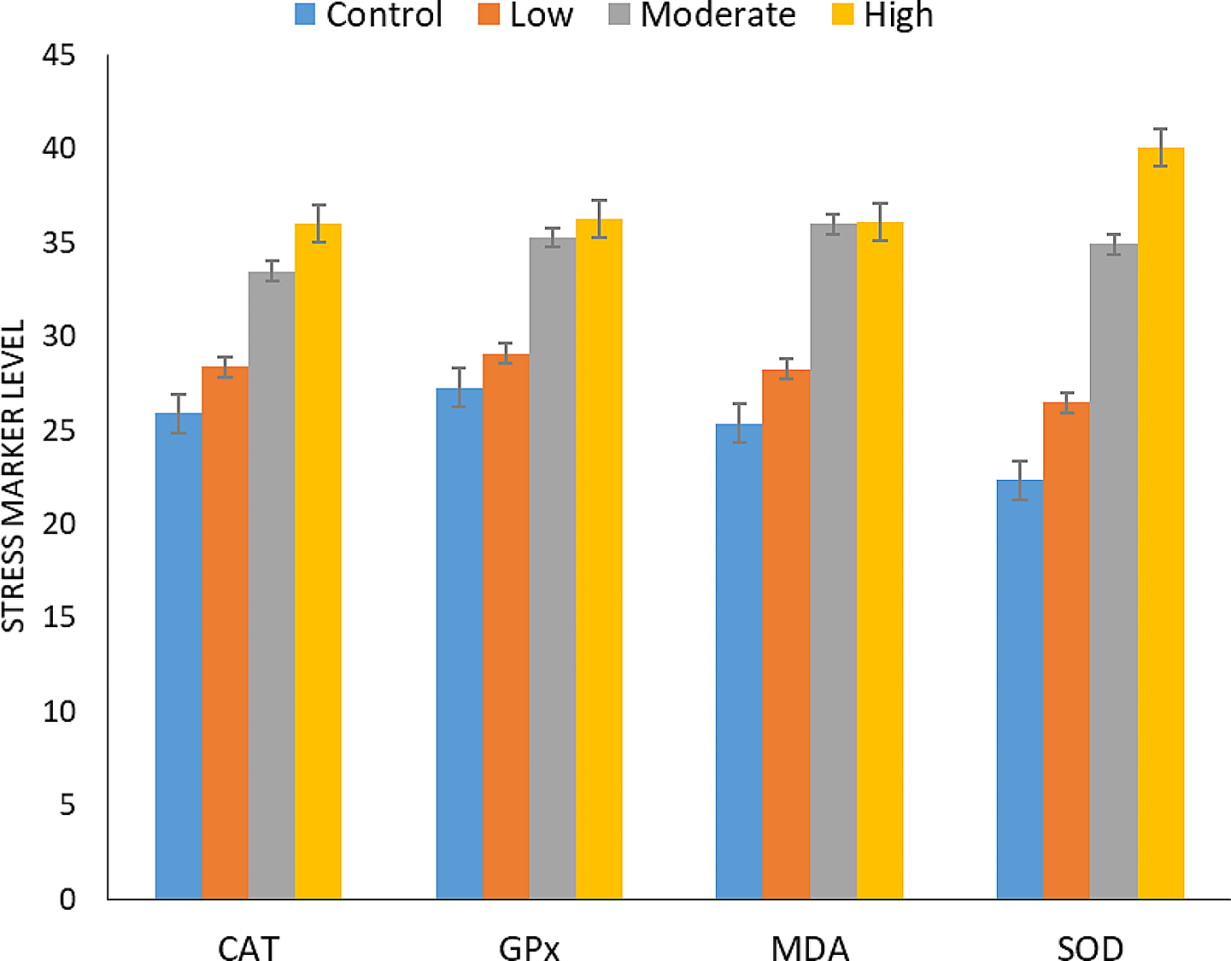
Markers of oxidative stress (CAT, GPx, MDA and SOD) of the infected horses

Investigating the relationship between the levels of parasitemia on the elevation of the levels of some markers of oxidative stress (CAT, GPx, MDA and SOD) revealed that there is a direct relation between the degree of elevation of these markers and the increase in the mean parasitemia in the infected horse in comparison with the control healthy one. The difference is high significance (*P* ≤ 0.0001) concerning the moderate and high levels of infection than in the low one in comparison with the control healthy one (Fig. 5; Table 7).

**Table 7:** Changes in some of the oxidative stress markers of the infected horses

Level of infection	CAT	GPx	MDA	SOD
Low	28.36 ± 0.98^bcd^	29.1 ± 0.44^bcd^	28.26 ± 2.05^bcd^	26.5 ± 1.02^bcd^
Moderate	33.49 ± 2.04^acd^	35.26 ± 2.12^ad^	35.9 ± 1.12^ad^	34.91 ± 0.63^acd^
High	36.03 ± 1.42^abd^	36.25 ± 1.70^ad^	36.12 ± 1.75^ad^	40.11 ± 3.22^abd^
Control	25.89 ± 0.53^abc^	27.26 ± 1.32^abc^	25.37 ± 0.85^abc^	22.34 ± 2.09^abc^
*P*-value	*P* ≤ 0.0001			

Data represented as the mean ± standard deviation (µg/ml), value of different letters per column are statistically significant at *P* ≤ 0.0001

### Effect of *T. equi* infection on some cytokines parameters (gene expression analysis)

**Fig. 6:**
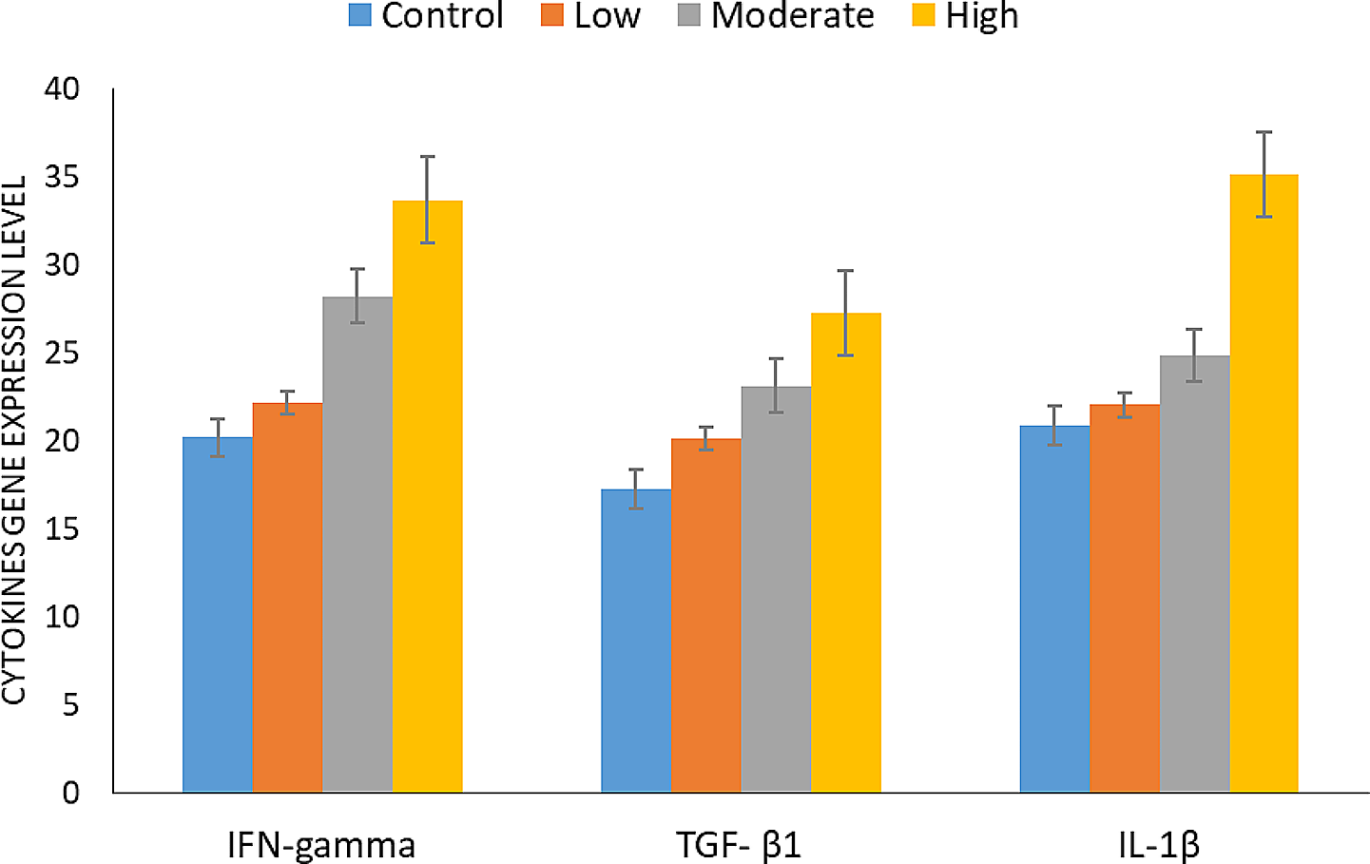
Analysis of gene expression of horses

The data in Table (8) & Fig. (6) revealed a marked significant increase in the level of different estimated cytokines parameters (IFN-gamma, TGF- β_1_ and IL-1β) genes in infected horses in comparison with the control non-infected animals. There is a direct relation between the increase in parasitemia and the increase in the level of IFN-gamma and IL-1β gene elevation in infected equines in comparison with healthy non-infected control horses.

**Table 8:** Analysis of gene expression of horses

Level of infection	IFN-gamma	TGF- β_1_	IL-1β
Low	22.17 ± 1.35^bcd^	20.13 ± 1.48^bcd^	22.03 ± 1.5^bc^
Moderate	28.22 ± 2.12^acd^	23.13 ± 1.38^acd^	24.86 ± 2.01^ad^
High	33.69 ± 1.32^abd^	27.23 ± 1.27^abd^	35.15 ± 1.5^ad^
Control	20.18 ± 0.57^abc^	17.25 ± 1.19^abc^	20.85 ± 0.99^bc^
*P*-value ≤ 0.0001	*P* ≤ 0.0001		*P* ≤ 0.01

Data represented as the mean ± standard deviation (µg/ml), value of different letters per column are statistically significant at *P* ≤ 0.0001

## Discussion

In the current study, 75 horses were assessed, and the infestation was shown by two tick genera, three species: *Hyalomma* (*H. marginatum* and *H. anatolicum*) and *Rhipicephalus* (*R. annulatus*). These species were identified based on their known morphological characteristics and phylogenetic analysis of the COXI gene, a relevant marker for tick species identification. The identified tick species were verified through GenBank BLAST using the GenBank database. The percentage distribution of the three collected tick species from infected equines, determined by sequencing the COXI genes, was *R. annulatus* (55.3%), *H. marginatum* (25.5%), and *H. anatolicum* (19.2%). These were assigned the accession numbers OR064161, OR067911, and OR187727, respectively, and were identical to tick species in the GenBank database. *T. equi* vector identification has been previously reported by Peckle et al. (2018). *Rhipicephalus* was the most frequently diagnosed genus in this study, known to pose significant health risks to infected equines in the Middle East and North Africa (Abdullah et al. 2022).

Equine theileriosis is challenging to diagnose in veterinary practice because microscopic examination of suspicious blood smears is often inaccurate, especially when there is low parasitemia. This investigation demonstrated PCR identification of the *T. equi* 18 S rRNA gene in adult ticks extracted from infected animals, showing a prevalence of 43.86% (386/880), while it was 50.7% (38/75) in horse blood samples displaying disease manifestations. These results align with the findings of Salim et al. (2008), Saleem and Al-Samarai (2018), Aziz et al. (2019), Bhoora et al. (2020), and Al-Rammahi et al. (2020). However, these results disagree with Ros-García et al. (2013), who reported a 12.5% prevalence rate in horses. This study is the first in Egypt to demonstrate the accurate determination of *T. equi* tick vectors through genotypic identification of the parasite within the ticks, comparing it to that diagnosed in the blood of infected horses. The obtained sequence was deposited in GenBank under the accession number OR068139.

Using COXI gene sequences, BLAST analysis verified the identification of tick species for two genera, *Rhipicephalus* and *Hyalomma*, showing results equivalent to those found in the GenBank database. A phylogenetic study indicated that the COXI gene is a sensitive and reliable identifier for tick species (Al-Hosary et al. 2021). The COXI locus sequence of *R. annulatus* showed higher nucleotide similarity with sequences reported by Shanan et al. (2017) from Iraq and Krishnamoorthy et al. (2021) from India, while it showed lower nucleotide similarity with *Rhipicephalus microplus* from Thailand and China (Rangubpit et al. 2023; Jin et al. 2023). *H. anatolicum* examined in this study was compatible with COXI sequences from Pakistan and Egypt (Sultan et al. 2022; Ashour et al. 2023a). For *H. marginatum*, the sequence was very similar to strains isolated from France and the Czech Republic (Vial et al. 2016; Lesiczka et al. 2022).

BLAST analysis of *T. equi* amplified fragments revealed sequence identities of 99–100% with previously published sequences; however, it is essential to note that the analysis was based on the 18 S rRNA gene. According to the phylogenetic analysis, the sequences showed higher nucleotide similarity with those reported by Rar et al. (2018), Elsawy et al. (2021), and Chen et al. (2022) from Russia, Egypt, and China, respectively.

The current study observed significant changes in blood markers of oxidative stress in horses with spontaneously occurring *T. equi* infections. These findings are consistent with the host’s efforts to develop intricate mechanisms in response to parasite infection, including generating reactive oxygen species (ROS) and/or nitrogen species (RNS). Superoxide dismutase (SOD) produces hydrogen peroxide, which, as a substrate for catalase (CAT), another antioxidant enzyme, may be harmful to cells. The investigation revealed elevated CAT, GPx, MDA, and SOD activity in infected horses compared to healthy ones. Oxidative stress in infected animals arises from an imbalance between increased blood markers of oxidative stress and the body’s scavenging mechanisms. Erythrocyte peroxidation, typically associated with the pathogen in hemoprotozoan infections, occurs particularly when ROS activity exceeds the antioxidant system’s capacity. Recent research on blood parasites has highlighted that oxidative damage caused by lipid peroxidation of red blood cells (RBCs) can lead to anemia (Omar et al. 2015). Esmaeilnejad et al. (2014) noted that hosts produce ROS to combat invasive infections, which can result in oxidative stress during parasitic illnesses. While these reactive chemicals have a defensive function, they cannot distinguish between infectious pathogens and host cells. The host activates defense systems, including antioxidant defenses, in certain situations. Oxidative stress can occur if ROS overwhelm the host (Rahal et al. 2014). Overproduction of ROS can lead to oxidative changes in DNA, proteins, and lipids, which are essential components of cells. ROS-induced lipid peroxidation of cell membranes can result in the loss of selective permeability and increased damage. Oxidative DNA damage may cause mutations, replication errors, genomic instability, and cell death, while oxidative protein damage can lead to the loss of metabolic activities (Klaunig et al. 2010).

Antioxidants, like glutathione peroxidase (GPX) and SOD enzyme activity, play a crucial role as the rate of parasitemia increases with their presence. Specifically, SOD is an efficient catalyst and exhibits high resistance to oxidative stress (Nazarizadeh and Asri-Rezaie 2016).

The current work, which examines the function of oxidative stress in *T. equi* pathogenesis in infected horses, shows that *T. equi* increases MDA concentrations via enhancing lipid peroxidation. Previous research indicates that this rise causes cell membrane damage linked to increased free radical generation. These results are consistent with Deger et al. (2009) in horses infected with *B. caballi*. Furthermore, Esmaeilnejad et al. (2014) have observed in ruminants that piroplasmosis and theileriosis are linked to elevated MDA concentrations, suggesting that heightened lipid peroxidation could be the primary mechanism causing piroplasmosis-induced cell membrane damage. The peroxidation of polyunsaturated fatty acids induced by ROS can increase membrane permeability and stiffness, potentially leading to harmful outcomes such as hemoglobin release and membrane disruption. The enhanced lipid peroxidation resulting from increased free radical antioxidants may be linked to the hemoglobinuria observed in infected horses in the current investigation. This observation is supported by earlier research showing a positive association between lipid peroxidation and the progression of anemia (Gopalakrishnan et al. 2015). This explanation clarifies the cause of anemia associated with *T. equi* infection due to the small size and slow or non-division of gametocytes in RBCs rather than the direct destruction of RBCs. This differs from the cause of hemoglobinuria in high infections with larger *Babesia* species like *B. caballi*, which directly destroy infected RBCs during their schizogony stages. Therefore, oxidative damage may play a significant role in the anemia pathophysiology in horses infected with *T. equi* (Salem & El-Sherif 2015).

The study revealed a significant (*P* ≤ 0.0001) elevation in the levels of various cytokine parameters (IFN-gamma, TGF-β1, and IL-1β) genes in horses infected with *T. equi* compared to non-infected control animals. This elevation was directly correlated with the level of parasitemia in infected horses, marking the first study in Egypt to investigate *T. equi* cytokine parameters, specifically focusing on gene expression of IFN-gamma, TGF-β1, and IL-1β in naturally infected horses. These findings align with previous research on babesiosis in cattle and mice, indicating a high IFN-gamma expression during the disease’s acute phase (Aziz et al. 2014).

Moreover, TGF-β1 mRNA levels were higher in *T. equi*-infected animals compared to non-infected ones, consistent with studies by Sasindran and Torelles (2011) and Maia and Campino (2012), which reported elevated TGF-β1 mRNA levels in bacterial and intracellular parasitic infections. Additionally, IL-1β mRNA levels were higher in infected animals than in healthy ones, in line with Aziz et al. (2014), who described IL-1β’s role in *T. equi* infection. The positive regulation observed between IL-1β and TGF-β1 suggests that IL-1β may be up-regulated in response to TGF-β1 up-regulation (Ganesh et al. 2011).

## Conclusion

The current study established an association between anemia, intermittent fever, and enlarged lymph nodes with a hard tick infestation in summer, indicating piroplasmes infection in equines. The study achieved genotypic identification of the *T. equi* tick vector to the species level using COXI-based molecular methods. Additionally, it identified the parasite in equine blood and infected vectors based on the amplification of the 18 S rRNA gene, marking the first time such genotypic identification has been accomplished in Egypt.

*T. equi* infection in equines triggers a specific and significant (*P* ≤ 0.0001) elevation in several oxidative stress markers (CAT, GPx, MDA, and SOD) and leads to an increase in serum cytokine parameters (IFN-gamma, TGF-β1, and IL-1β) genes. The elevation of these markers directly correlates with the level of parasitemia, implying their significant role in the pathophysiology of equine theileriosis.
